# Polyunsaturated fatty acids: any role in rheumatoid arthritis?

**DOI:** 10.1186/s12944-017-0586-3

**Published:** 2017-10-10

**Authors:** Luca Navarini, Antonella Afeltra, Gabriele Gallo Afflitto, Domenico Paolo Emanuele Margiotta

**Affiliations:** Unit of Allergology, Immunology, Rheumatology, Department of Medicine, Università Campus Bio-Medico di Roma, via Álvaro del Portillo 21, 00128 Rome, Italy

## Abstract

**Background:**

Polyunsaturated fatty acids (PUFAs) are members of the family of fatty acids and are included in the diet. Particularly, western diet is usually low in n-3 PUFAs and high in n-6 PUFAs. PUFAs play a central role in the homeostasis of immune system: n-6 PUFAs have predominantly pro-inflammatory features, while n-3 PUFAs seem to exert anti-inflammatory and pro-resolving properties. Rheumatoid arthritis (RA) is a chronic inflammatory arthritis in which many inflammatory pathways contribute to joint and systemic inflammation, disease activity, and structural damage. Research on PUFAs could represent an important opportunity to better understand the pathogenesis and to improve the management of RA patients.

**Methods:**

We searched PubMed, Embase, EBSCO-Medline, Cochrane Central Register of Controlled Trials (CENTRAL), CNKI and Wanfang to identify primary research reporting the role of n-3 PUFAs in rheumatoid arthritis both in humans and in animal models up to the end of March 2017.

**Results:**

Data from animal models allows to hypothesize that n-3 PUFAs supplementation may represent an interesting perspective in future research as much in prevention as in treating RA. In humans, several case-control and prospective cohort studies suggest that a high content of n-3 PUFAs in the diet could have a protective role for incident RA in subjects at risk. Moreover, n-3 PUFAs supplementation has been assessed as a valuable therapeutic option also for patients with RA, particularly in order to improve the pain symptoms, the tender joint count, the duration of morning stiffness and the frequency of NSAIDs assumption.

**Conclusions:**

n-3 PUFAs supplementation could represent a promising therapeutic option to better control many features of RA. The impact of n-3 PUFAs on radiographic progression and synovial histopathology has not been yet evaluated, as well as their role in early arthritis and the combination with biologics.

## Background

Rheumatoid Arthritis (RA) is a chronic inflammatory arthritis. In recent years, great strides have been made towards a deeper understanding of pathogenesis, early diagnosis and treatment of this disease. Particularly, in the last 20 years the optimization in the use of the Disease-Modifying Antirheumatic Drugs (DMARDs) and the advent of biologics and small molecules, like Janus kinase (JAK) inhibitors, have profoundly improved the clinical outcomes of patients with RA [1]. Furthermore, the definition of a treat-to-target management strategy allows to achieve remission and to stop the progression of structural damage bone in a significant percentage of patients. However, a relevant part of patients still does not reach the therapeutic objectives [2, 3]. For example, sustained and drug-free remission, cardiovascular and pulmonary comorbidities management, and optimization of cost-effectiveness of anti-rheumatic treatments remain unmet needs [4, 5]. Therefore, a better understanding of the pathological mechanisms of this disease and the development of new pharmacological approaches are an important challenge.

The relationship between polyunsaturated fatty acids (PUFAs) and rheumatoid arthritis has been extensively evaluated in many studies, but a lot of questions remain unanswered. Their impact on immune system and inflammatory diseases is charming indeed: n-6 PUFAs exert mostly pro-inflammatory features, while n-3 PUFAs have anti-inflammatory and pro-resolving effects. Thus, many researches have investigated a possible role of n-3 PUFAs supplementation as a preventative strategy for the development of arthritis and/or a low cost treatment to be added to conventional therapy in order to better achieve a comprehensive disease control.

In this review, we aim to discuss the state of art of knowledge about the use of PUFAs supplementation in the management of patients with RA. We searched PubMed, Embase, EBSCO-Medline, Cochrane Central Register of Controlled Trials (CENTRAL), CNKI and Wanfang up to March 31, 2017. The following key words were included: n-3 PUFAs, eicosapentaenoic acid or EPA, docosahexaenoic acid or DHA, arthritis, rheumatoid arthritis. We examined the bibliographies of relevant articles for additional publications.

## PUFAs: structure and effects on immunity

PUFA are fatty acids characterized by the presence two or more double bonds [6]. The location of the last double bond relative to the terminal methyl-end of the molecule allows the classification in n-3 or n-6 PUFAs. PUFAs constitute an important component of the diet: particularly, western diet is usually low in n-3 PUFAs, like alpha-linolenic acid (ALA; 18:3n-3), eicosapentaenoic acid (EPA; 20:5n-3), docosapentaenoic acid (DPA; 22:5n-3), and docosaheaxaenoic acid (DHA; 22:6n-3), and high in n-6 PUFAs, like linoleic acid (LA; 18:2n-6) or arachidonic acid (ARA; 20:4n-6). The major dietary source of n-3 PUFAs is fish, and particularly fish oil (for EPA and DHA), and plants (for ALA), while the major dietary sources of n-6 PUFAs are vegetable oils and animal sources [7]. Increasing evidence is showing that an adequate dietary intake of EPA and DHA is important for many physiological processes; the conversion of ALA into EPA and DHA is not very efficient in humans and cannot compensate an intake deficit [8, 9]. Moreover, in western diet, the balance between n-6 and n-3 PUFAs intake is generally dysregulated in favour of n-6 one [7].

Particularly, PUFAs play a pivotal role in the immune system homeostasis; notably, n-6 PUFAs mostly display pro-inflammatory functions. In fact, the major part of the ARA-derived compounds act as pro-inflammatory mediators: for example, from ARA, through the action of cyclooxygenase (COX), prostaglandins (PG) and thromboxanes (TX) are produced, whereas leukotrienes (LT) are obtained by the action of lipoxygenase (LOX) [10]. Pro-inflammatory PGs act in a wide range of process like vascular permeability, tissue regeneration, fibrosis, pain perception, adhesion molecules expression and leukocytes migration in inflammatory tissues (e.g. in an animal model, the dietary supplementation of n-3 PUFAs can decrease the adhesion molecules expression in endothelium) [11]. LTs play as pro-inflammatory mediators, too; for example, they increase COX-2 expression in mast cells with subsequent PGD_2_ production [12, 13]. Even endocannabinoids (eCBs), such as *N-*arachidonoylethanolamine (anandamide, AEA) and 2-arachinoylglicerol (2-AG), lipid molecules which interact with specific G protein-coupled type-1 (CB_1_) and type-2 (CB_2_) cannabinoid receptors, are arachidonic acid (AA) derivatives [14]. Particularly, eCBs act as regulator of the immune system homeostasis: in, fact, AEA seem to have anti-inflammatory properties, while 2-AG exhibits both pro-inflammatory and anti-inflammatory functions; CB_2_, more expressed in immune cells than CB_1_, seems to have a major role in mediating the eCBs effects on immune system [15].

Contrariwise, n-3 PUFAs show anti-inflammatory properties. Firstly, n-3 PUFAs intake tends to reduce the amount of AA in immune cells and thus to reduce AA derivatives pro-inflammatory activities [16]: for example, EPA intake of 2.7 g/day is able to reduce PGE_2_ production in vivo [17]. EPA could also become a substrate for COX but, in this case, its derivatives (e.g. 3-series PGs and TXs and 5-series LTs) will exhibit a lower biological and inflammatory potency than ARA-derived eicosanoids [16]. For example, PGE_3_ seems to be 50–80 times less potent than PGE_2_ towards the EP1, EP2, EP3 and EP4 receptors [18]. n-3 PUFAs reduce AEA and 2-AG concentrations - which is probably due to the reduction of AA in cell membranes -, but they increase the production of endocannabinoids with EPA or DHA in their structure, like docosahexaenoyl ethanolamide and eicosapentanoyl ethanolamide [19, 20], which express anti-inflammatory effects in vitro [21, 22]. The relationship between n-3 PUFAs and cytokines production is quite important as well: on animal models, EPA and DHA dietary supplementation is associated with a lower production of TNF-α, IL-1β, and IL-6 and to an increased level of the anti-inflammatory cytokine IL-10, after endotoxin injection. Especially when >2 g/die of EPA + DHA are administrated, they can reduce TNF-α, IL-1β, and IL-6 production by monocytes and macrophages stimulated by endotoxin [16]. Supplementation of EPA and DHA has been shown to reduce the expression of adhesion molecules on immune cells and on endothelium both in animal models and in humans [16], via NFκB, PPAR-γ and GPR120. Moreover, several in vitro studies have demonstrated a reduction of matrix metalloproteinases (MMPs) production in myocytes [23], fibroblasts [24], keratinocytes [25], macrophages [26], and chondrocytes [27]; nevertheless, in vivo data about n-3 PUFAs supplementation and MMPs production are still limited and often conflicting with in vitro studies [16]. The anti-inflammatory properties of n-3 PUFAs on T lymphocytes are particularly interesting. EPA and DHA can reduce T-cell proliferation and the production of IL-2, in vitro [28, 29]; these data are confirmed in humans treated with high EPA + DHA intake [30]. The effects of n-3 PUFAs on Th17 lymphocytes is intriguing: in *fat-1* mice (which are able to convert n-6 in n-3 PUFAs), after colitis induction, Th17 cell number in lymphoid tissues and related cytokines in colonic mucosa were lower than wild-type mice [31].

EPA and DHA exhibit not only anti-inflammatory properties, but also help to restore the homeostasis of tissues after inflammation: some of their metabolites are considered specialized pro-resolving mediators [32]. From this point of view, the metabolism of EPA can lead to the formation of Resolvin E (RvE) series and the metabolism of DHA can lead to the formation of Resolvin D (RvD) series, maresins, and protectins, which are able to stimulate resolution mechanisms of inflammation [15, 33–36]. RvE1, RvD1, and protectin D1 are able to prevent transendothelial migration of neutrophils; RvD1 and protectin D1 can inhibit IL-1β production; protectin D1 also inhibits TNF-α production [16].

PUFAs may have a role in autotaxin (ATX)/lysophosphatidic acid axis (LPA). ATX is a lysophospholipase D, which converts lysophosphatidylcholine (LPC) to LPA [37, 38]. ATX synthesis can be up-regulated in inflammatory tissues, leading to the production of LPA, which have an important role in cell growth and motility; ATX/LPA axis could represent a key pathway in immune cells and inflammatory disorders [39]. Notably, in rheumatoid arthritis synovial fibroblasts TNF-α can induce ATX expression [40] and increasing evidence has shown that LPA1 receptor could represent an intriguing therapeutic option [41, 42]. Of interest, in a cancer model carboxyl group-containing PUFAs inhibited LPA-induced calcium/RhoA signalling pathway [43].

**Fig. 1:**
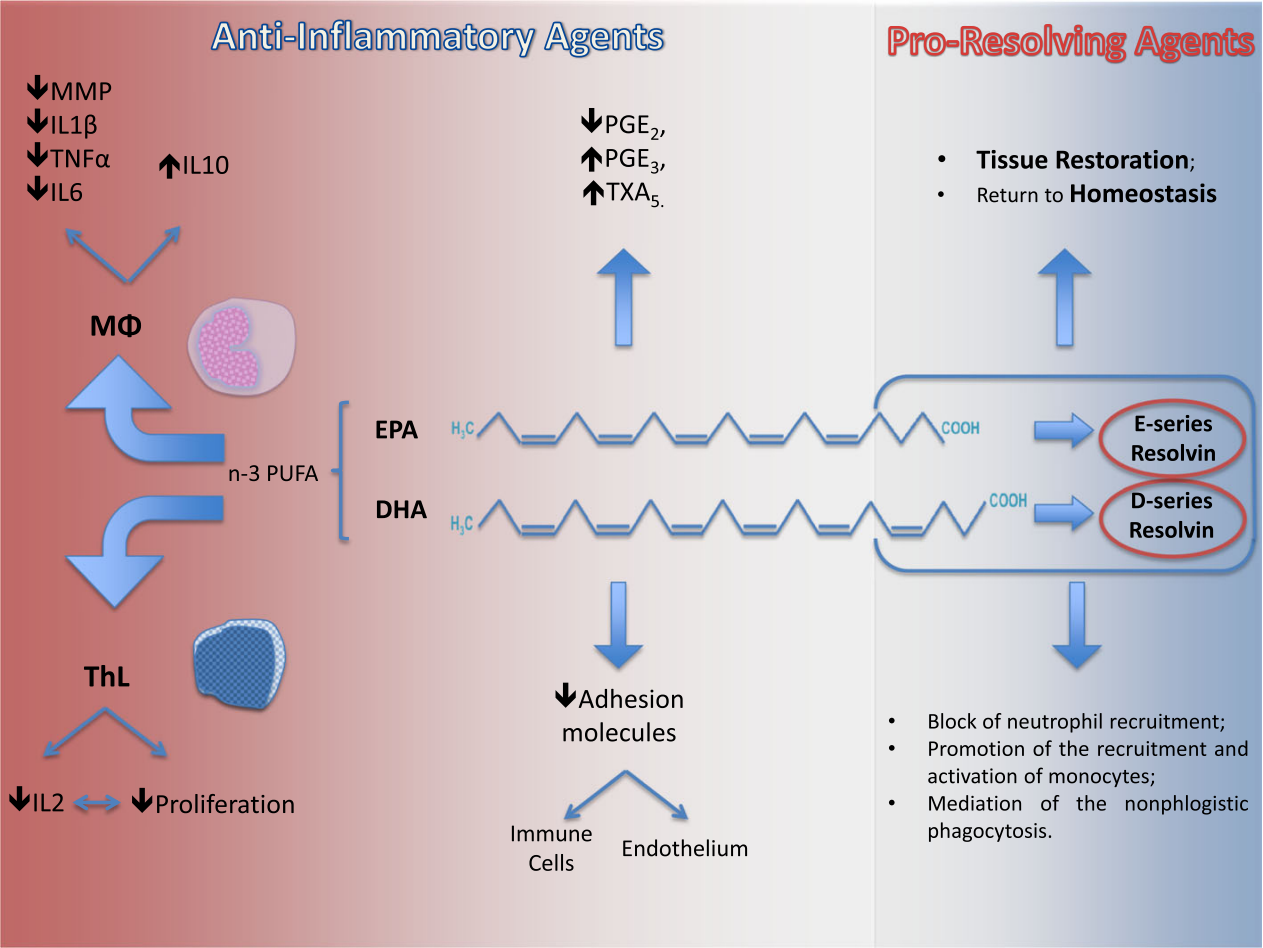
n3 PUFAs act, directly and indirectly, both as anti-inflammatory and as pro-resolving mediators. On MFs, they are able to inhibit the synthesis and secretion of MMPs, IL-1β, TNF-α, and IL-6, thus promoting IL-10 secretion. On T H Ls, they exhibit anti-proliferative features, mediated by the inhibition of IL-2 secretion. Moreover, they compete with n-6 arachidonic acid, determining a reduction in lipid derived pro-inflammatory compounds production. In addition, the anti-inflammatory action exerted by EPA and DHA is mediated by a reduction in the expression of adhesion molecules both on immune cells and on endothelium. On the other hand, pro-resolving mediators, such as E-series and D-series resolvins, produced by the metabolism of EPA and DHA, are responsible for tissue restoration and return to homeostasis blocking neutrophil recruitment, promoting the recruitment and the activation of monocytes and mediating non phlogistic phagocytosis. PUFA, Polyunsaturated fatty acids; EPA, Eicosapentaenoic acid; DHA, Docosahexaenoicacid; MFs, Macrophages; T H Ls, T-helper Lymphocytes; PG, Prostaglandin; TX, Thromboxane; IL, Interleukin

Anti-inflammatory and pro-resolving features of n-3 PUFAs are summarized in Fig. 1.

## PUFAs and animal models of RA

A first report demonstrated that in collagen-induced arthritis (CIA) fish oil, when administered before the immunization, can delay the onset of symptoms, to decrease the incidence and to reduce the severity of the disease in comparison with corn-oil. These effects seemed more pronounced in female than in male [44]. In the same model, DHA administration, when begun 4 weeks prior to the arthritis induction, reduces the incidence and the severity of arthritis, while DHA/EPA combination does not show beneficial effects. DHA is also able to reduce pannus formation, cartilage destruction, bone damage, pro-inflammatory cytokines levels and anti-collagen antibodies, while DHA/EPA combination does not show any beneficial effect on synovial histopathology and anti-collagen antibodies production [45]. Recently, another study evaluated the impact of fish or krill oil on collagen-induced arthritis. Krill oils contain n-3 PUFAs but in the form of phospholipids, while fish oil mostly in the form of either triacylglycerol or fatty acid ethyl esters. In this study, krill or fish oil were administered before the induction of arthritis. Both fish and krill oil supplementation leads to a decreased incidence of arthritis, reduced severity of arthritis and decreased synovial histopathological findings [46]. In the same mouse model, it was established that extra virgin olive oil (EVOO)-polyphenol extract (PE) administration is able to decrease joint swelling, bone destruction and cartilage degradation reducing pro-inflammatory cytokines production (like IL-1β, TNF-α, and IL-6), COX-2 expression, and NF-κB activity [47]. Particularly, a single EVOO-polyphenol administration, HTy-Ac, is able to prevent inflammation of the joints, reducing serum IgG_1_ and IgG_2_ serum levels, MMP-3 and inflammatory cytokines secretion, and JAK-STAT, MAPKs, and NF-κB activation [48]. Of interest, also the administration *N*-plamitoylethanolamine (PEA), a fatty acid amide belonging to the family of *N*-acylethanolamines (NAEs) which is considered as an eCB-like compound [49], and the flavoinoid luteolin is able to reduce the severity of CIA in mice [50]. Also trans-10,cis-12 (t10c12) conjugated linoleic acid (CLA), which is present in corn, sunflower, and fassflower oil, reduces CIA in mice when fed a diet containing 0.125% of this compound, a very high dosage which is nearly impossible to administer to humans [51].

In another study, Lew/SSN rats were fed with different ratios of EPA and DHA before the induction of streptococcal cell wall (SCW) arthritis. EPA and DHA could reduce the severity of arthritis symptoms, but synovial histopathological findings seemed to improve more with EPA than with DHA [52].

Dietary intake of n-3 and n-6 PUFAs is important also for bone metabolism. The long-term effect of n-3 and n-6 PUFAs supplementation in a mouse model of rheumatoid arthritis (MRL/lpr female mouse) was evaluated on bone mineral density (BMD) and metabolism. In this model, fish oil supplementation leads to a higher BMD and to a lesser extent synovial inflammation than corn oil supplementation. Furthermore, fish oil intake decreased RANKL and increased osteoprotegerin gene expression [53].

Castillero E. and co-authors studied the effects of EPA on skeletal muscle wasting during arthritis. First of all, arthritis in rats was induced by Freund’s adjuvant injection and then a supplementation of EPA was started. When receiving EPA, rats showed lower arthritis symptoms and an increase in relative gastrocnemius weight. EPA supplementation was able to reduce TNF-α, Atrogin-1, and MuRF1 gene expression. Atrogin-1 and MuRF1 are “atrogenes” genes and they are increased in muscle wasting conditions, such as cancer or diabetes [54]. In addition, n-3 monoglycerides, named eicosapentaenoic acid monoglyceride (MAG-EPA), docosahexaenoic acid monoglyceride (MAG-DHA), and docosapentaenoic acid monoglyceride (MAG-DPA) have been evaluated as a potential therapy in the same rat model. MAG-DPA and MAG-EPA can reduce hind paw thickness, prevent disease progression and reduce cytokines (IL-17A, IL-1β, IL-6, TNF-α) and metalloproteinase (MMP-2 and MMP-9) production, while MAG-DHA, after an initial efficacy, does not shown any benefit in later stages of the disease [55]. In the same murine model, the administration of fish oil preparation (FOP) (75, 150, and 300 mg/kg for 21 days after the induction of the arthritis) is able to decrease the migration, adhesion, and recruitment in the joints of leukocytes [56]. Furthermore, the administration of DHA (both orally and intra-articularly) reduces the nociception and joint swelling in mice after the injection of Complete Freund’s Adjuvant (CFA) in the right knee [57].

*fat-1* mice have a decreased n-6/n-3 PUFA ratio because of their ability to convert n-6 in n-3 PUFAs [58]. In a recent study, *fat-1* mice were injected with *K/BxN* serum in order to induce arthritis. In this model, *K/BxN* serum-transfer arthritis severity, as well as TNF-α, IL-1β, IL-6, MCP-1, IFN-γ, MMP-3, MMP-13 and RANKL production, and p38 MAPK and JAK-STAT-3 pathway activation are lower in *fat-1* mice. Moreover, activity of the osteoclasts seems to be reduced in a low n-6/n-3 PUFA ratio environment. These findings suggest that n-3 supplementation and n-6 reduction could represent a feasible therapeutic option for *K/BxN* arthritis [59].

Overall, data from animal models provide evidence that PUFAs administration could be beneficial in arthritis. Particularly, n-3 PUFAs from fish oil could have a preventive role in the development of arthritis and are able signs and symptoms of joint disease. These effects are obtained through the reduction of pro-inflammatory cytokines and metalloproteinases production and the decrease of leukocytes migration. It is not well established whether these anti-inflammatory effects are to be attributed predominantly to DHA or EPA: at present, different animal models provide different results. n-3 PUFAs supplementation could also prevent bone and cartilage destruction during inflammatory arthritis, mainly decreasing osteoclasts activation, and reduce joint pain [60]. But also other compounds showed to provide some beneficial effect in experimental arthritis, for example EVOO-PE, HTy-Ac, PEA, and luteolin.

## Clinical trials in RA: dietary PUFAs supplementation

Results of clinical trials on n-3 PUFAs and RA were summarized in Table 1.

**Table 1:** Results of clinical trials on n-3 PUFA and RA

Author, Year	Type of study	N	Intervention	Results	References
Kremer JM et al., 1985	Db-CT	17 exp. group 20 ctr	Exp group: diet high in PUFAs and low in saturated fat, with a daily supplement (1.8 g) of EPA. CTR: diet with a lower PUFAs ratio and a placebo supplement.	At week 12: Exp Group: improvement in morning stiffness and TJC. 2 months after stopping diet: deterioration in EGA, PGA, pain and TJC	[61]
Kremer JM et al., 1987	dd-CT with cross-over design	21 exp. group 19 ctr	14-week treatment periods and 4-week washout periods Exp group: a daily dosage of 2.7 g of EPA and 1.8 g of docosahexenoic acid CTR: identical-appearing placebos	At week 14: Exp group: improvement of mean time to onset of fatigue and TJC Persistent of effect after 4 weeks washout	[62]
Kremer JM et al., 1990	Db -RCT	20 Exp group A 17 Exp group B 12 Exp group C	24 week trial: Exp Group A: 27 mg/kg EPA and 18 mg/kg DHA (low dose regimen) Exp Group B: 54 mg/kg EPA and 36 mg/kg DHA (high dose regimen) Exp group C: olive oil capsules containing 6.8 g of oleic acid	At week 24: Improvement in TJC and SJC in group A and B (low and high dose group) Improvement of SJC at week 12 only in group B. Reduction of neutrophil LTB in both A and B group. Reduction of macrophage IL1 only in group B.	[65]
Van der Tempel H et al., 1990	Db-RCT	16 RA patients	12 week trial with crossover design Exp group: fractionated fish oil fatty acids Control group: fractionated coconut oil	At week 12: Improvement of SJC and duration of morning stiffness in Exp group. Reduction of neutrophil LTB4 in Exp group.	[67]
Espersen JT et al., 1992	Db-RTC	32 active RA patients	12 week trial: Exp group: dietary supplementation with n-3 fatty acids (3.6 g per day) Control group: placebo	At week 12: Reduction of IL1beta in Exp group Improvement in Ritchie index in Exp group-	[69]
Magarò M et al., 1992	RCT	10 Exp group 10 control group	45 days trial Exp group: dietary supplementation of 1.6 g of EPA and 1.1 g of DHA Control group: continued usual diet.	At 45 days: Reduction of neutrophils chemiluminescence in Exp group. Reduction of Rithie index, morning stiffness and ESR in Exp group.	[70]
Nielsen GL et al., 1992	Db-RCT	51 RA patients	12 week trial: Exp group: dietary supplementation of 3.6 g of n-3 PUFAs Control group: supplementation with fat composition as the average	At week 12: Improvement of morning stiffness and TJC in Exp group	[71]
Kjeldsen-Kragh J et al., 1992	Db-trial	67 active RA	29 weeks trial: Placebo Group 1: corn oil 7 g/day for 16 weeks, and naproxen 750 mg/day for 10 weeks followed by a stepwise reduction to 0 mg/day during the following 3 weeks Exp Group 2: 3.8 g of EPA + 2.0 g of DHA and naproxen 750 mg/day for 16 weeks Exp Group 3: EPA + DHA as Group 2 and naproxen as Group 1.	At the end of the trial: - Improvement of morning stiffness duration, PGA and EGA in Exp group 2.	[72]
Geusens P et al., 1994	Db-RCT	90 active RA: 19 Exp group A 21 Exp group B 20 Control group	52 weeks trial: Exp group A: 2.6 g of n-3 PUFAs Exp group B: 1.3 g ofn-3 PUFAs +3 g of olive oil, Control group: 6 g of olive oil	Significant reduction in PGA after 3 months, maintained up to 12 months in Exp A group. Higher proportion of patients in Exp group A with reduction in PGA, pain score and the assumption of NSAID and/or DMARDs.	[66]
Kremer JM et al., 1995	Db-trial	Active RA taking diclofenac 75 mg/twid 37 Exp group 29 Control group	48 week trial: - Diclofenac replaced with placebo at week 18 or 22 - fish oil replaced with corn oil at week 26 or 30 Exp group: 130 mg/kg/day of n-3 PUFAs until week 26. Than 9 capsules/day of corn oil Placebo group: 9 capsules/day of corn oil	At the first visit while taking diclofenac placebo (week 22 or 26): - Reduction of TJC, morning stiffness, PGA, EGA, pain index in Exp group - Reduction of IL1beta levels in Exp group 8 weeks after discontinuation of diclofenac: - Maintenance of TJC reduction in Exp group.	[73]
Nordstrom DC et al., 1995	Clinical Trial	22 Active RA	3 months trial: Exp group: alpha-LNA Control group: placebo	At the end of the trial: - No change in clinical and laboratory parameters - No change in EPA, DHA and A levels	[76]
Belch JJ et al., 1998	Db-CT	16 Exp group A 15 Exp group B 18 CTR	12 months of treatment followed by 3 months of placebo Exp goup A: 540 mg GLA/day (EPO), Exp group B: 15 patients 240 mg EPA and 450 mg GLA/day (EPO/fish oil) CTR inert oil (placebo).	At 12 months: subjective improvement of symptoms and reduction of NSAID in group A and B. After additional 3 month on placebo: relapse of symptoms in group A and B.	[63]
Volker D et al., 2000	Db-RCT	50 RA patients with n-6 PUFAs intake in background diet <10 g/day	15 weeks trial: Exp group: fish oil with 60% of n-3 PUFAs at dosage of 40 mg/Kg body weight Control group: placebo	At the end of the trial: Significant improvement in clinical variable in Exp group. Increase of EPA in plasma and monocyte lipids in Exp group	[68]
Remans PH et al., 2004	Db-RCT	66 RA patients	4 months trial: Exp group: dietary supplementation containing 1.4 g of EPA, 0.211 g of DHA, 0.5 g of gamma-LNA and micronutrients Control group: placebo	At the end of the trial: No change in clinical varable Increase in plasma EPA, DHA, vitamin E and decrease in AA in Exp group	[64]
Leeb BF et al., 2006	Open pilot study (one group design)	34 active RA (DAS28 > 4)	5 week study: 2 mL/kg (= 0.1–0.2 g fish oil/kg) fish oil emulsion intravenously on 7 consecutive days. Background therapy unchanged.	At the end of the study: - No safety issues - Overall reduction of DAS28 from baseline - Reduction of DAS28 > 0.6 in 41% of patients	[78]
Galarraga B et al., 2008	Db-RCT	97 RA	9 months trial. At 12 weeks, patients were instructed to gradually reduce, and if possible, stop their NSAID intake Exp group: 10 g of cod liver oil containing 2.2 g of n-3 PUFAs Control group: air-filled identical placebo capsules	At the end of the study: 39% of patients in the Exp group and 10% in the control group were able to reduce their daily NSAID requirement by >30%.	[74]
Dawczynski C et al., 2009	Db-RCT with cross over design	45 RA patients	2 investigation periods of 3 and an 2 months washout phase between the 2 periods. Verum: daily diet containing 40 g of fat (200 g of yogurt, 30 g of cheese, 20–30 g of butter). The milk fat was partially exchanged with special oil with high concentration of EPA and alpha-LNA. Overall n-3 PUFAs: alpha-LNA 1.1 g, EPA 0.7 g, DPA 1.1 g, DHA 0.4 g. Placebo: commercial dairy products with comparable fat contents	At the end of the 3 months treatment period: - improvement of lipid profile (increase of HDL and reduction of LpA) - Decrease in lipopolysaccharide-stimulated cylo-oxygenase-2 expression - Decrease of lymphocytes and monocytes blood count. At the end of the overall follow-up: - Reduce diastolic blood pressure	[108]
Bahadori B et al., 2010	Db-RCT	23 active RA patients	22 weeks trial: Exp group: 0.2 g of fish oil emulsion/kg infusion IV for 14 consecutive days followed by 20 weeks of 0.05 g of fish oil/kg capsule. Control group: 0.9% saline infusion IV for 14 consecutive days, followed by 20 weeks of paraffin wax capsule.	After 1 and to weeks of IV infusion: - reduction of SJC in the Exp group. At the end of the study: - reduction of SJC and TJC in the Exp group.	[79]
Proudman SM et al., 2015	RCT	Active Early RA: disease duration <12 months, DMARDs naïve 86 Fish oil group 53 controls	52 weeks trial: DMARD combination of MTX, sulphasalazine, hydroxychloroquine and leflunomide according to a predetermined algorithm based on disease activity and toxicity Fish oil group: 5.5 g/day of n-3 PUFAs (EPA + DHA) Control group: 0.4 g/day of n-3 PUFAs	At week 52: - Failure of triple DMARD therapy was lower in the fish oil group adjusted HR = 0.24 (adjusted for smoking history, shared epitope and baseline anti-cyclic citrullinated peptide) - The rate of first ACR remission was significantly greater in the fish oil group adjusted HR = 2.09.	[77]
Rajaei E et al., 2015	Db-RCT	60 active RA	12 weeks trial Exp group: 2 capsules/day of n-3 PUFAs (1.8 g of EPA + 2.1 g of DHA). Control group: placebo	At the end of the trial: - Improvement of EGA and PGA in the Exp group - Reduction of NSAID assumption in the Exp group	[75]

*EPO* evening primrose oil, *EPA* Eicosapentaenoic acid, *DHA* docosahexaenoic acid, *alpha-LNA* alpha-linoleic acid, *gamma-LNA* gamma-linoleic acid, *AA* arachidonic acid

The first clinical trial on n-3 PUFAs dietary supplementation in RA was reported by Kremer JM and co-authors, in 1985. The experimental group was treated with an experimental diet high in EPA (1.8 g/day). After 12 weeks authors reported an improvement on Tender Joints Count (TJC) and morning stiffness [61]. In 1987, the same group conducted a nonrandomized, double-blinded, placebo-controlled, crossover trial with 14-week treatment periods and 4-week washout periods. Twenty-one RA patients began with a daily dosage of 2.7 g of EPA and 1.8 g of DHA, while 19 RA patients began with identical-appearing placebos. The background diet was unchanged and treatment with non-steroidal anti-inflammatory drugs (NSAID), slow-acting antirheumatic drugs, and prednisone was allowed. In the experimental group, mean time to onset of fatigue improved by 156 min and number of tender joints decreased by 3.5. Neutrophil leukotriene B4 production was correlated with the decrease in the number of tender joints. The effects of fish oil supplementation persisted beyond the 4-week washout period [62]. Both studies are of historical importance and provided the first evidence in humans of effectiveness of n-3 PUFAs supplementation in RA, but they are limited by the small sample size and by the short duration of the analyses. Moreover, in both studies, the clinical impact of the treatment is limited to TJC.

In 1988, Belch JJ and co-authors tried to determine if evening primrose oil (EPO) or EPO/fish oil could replace NSAID treatment in RA. Unfortunately, this study is not properly relevant to the topic of the present review because of the low doses of EPA used (only 240 mg/day) [63]. Similarly, Remans PH and co-authors investigated the effect of dietary supplementation containing EPA, DHA, GLA and micronutrients in a double-blind RCT on 66 RA patients. At the end of the 4 months study, any change in clinical parameters was not observed [64]; However, it must be emphasized the low EPA doses in the dietary supplementation compared to the other studies.

The impact of dietary supplementation of n-3 PUFAs was extensively compared with other kinds of oils. In 1990, Kremer JM and co-authors compared the effect of dietary supplementation with 2 different dosages of fish oil and 1 dosage of olive oil in 24 weeks RCT. Twenty patients consumed a daily low dose dietary supplements of n-3 fatty acids, 17 patients ingested a daily high dose dietary supplements on n-3 fatty acids, and 12 patients ingested olive oil capsules. At week 24, significant improvement in TJC and swollen joint count (SJC) was observed in high and low dose group. A decrease in neutrophil LB4 production was noted in both n-3 fatty acids supplementation group, while only in high dose group macrophage IL-1 production decreased. Tritiated thymidine incorporation in peripheral blood mononuclear cells after stimulation with concanavalin A increased significantly in all 3 groups after 24 weeks, compared with baseline values [65]. The interest of this study is the comparison of high and low doses of fish oil supplementation. Moreover, in a sizable observation time, the impact of the treatment is not only TJC reduction but also improvement in SJC. However, no significant improvement from baseline was detected regarding patient evaluation of pain and global disease activity, an unexpected result in the context of the other data of this study. Unfortunately, in the context of patients reported outcomes (PROs), no quality of life assessment was performed. Geusens P and co-authors reported a long term double blind RCT including three group: daily supplementations with either 2.6 g of n-3 PUFAs, or 1.3 g of n-3 PUFAs +3 g of olive oil, or 6 g of olive oil alone. After 12 months, the proportions of patients who improved and of those who were able to reduce their concomitant anti-rheumatic medications were significantly greater with 2.6 g/day of omega 3 [66]. The added value of this study is the long-tern observation and the evidence that only high dose n-3 PUFAs supplementation can really lead to RA clinical improvement as well as to NSAID and DMARDs lowering. The results about olive oil dietary supplementation are still controversial. Van der Temple H and co-authors reported a 12 week RCT with a crossover design to evaluate the effect of dietary supplementation with fractionated fish oil fatty acids compared to placebo consisted of fractionated coconut oil. In the fish oil group was noted a significant improvement in SJC and early morning stiffness and a reduction of neutrophil LTB4 production at 12 week [67]. The impact of dietary supplementation on clinical parameters as well as on production of inflammatory mediators is an interesting value of this RCT. However, the crossover design could be limited by the short study duration. Wolker D and co-authors reported a double-blind RCT in RA patients whose n-6 PUFAs intake in the background diet was <10 g/day. Supplementation with fish oil was compared to olive/corn oil capsule supplement over a 15 weeks period. At the end of the study, clinical variables improved in fish oil group. Moreover, authors described an increase of EPA in plasma and monocyte lipids in the supplemented group [68]. The interest of this study is the inclusion criteria of the RA sample. However, the short duration could limit results interpretation.

As reported for mouse models, n-3 PUFAs supplementation could lead to decrease of inflammatory cells and mediators in human rheumatoid arthritis. For example, in 1992 Espersen GT and co-authors conducted a 12-week double-blind RCT evaluating the effect of n-3 PUFAs dietary supplementation on the level of cytokines and complement activation in plasma. To the treatment group was administrated a dietary supplementation with n-3 fatty acids (3.6 g per day). The IL-1β concentration in plasma was reduced significantly after 12 weeks of dietary supplementation with fish oil, whereas no significant changes were observed in the degree of complement activation and in TNF-α levels. Moreover, in the treatment group was described an improvement in the Ritchie’s articular index [69]. In a RCT on 20 active RA patients, in which 10 patients assumed dietary supplementation of fish oil and 10 patients continued their usual diet, Magarò M and co-authors demonstrated a progressive reduction of chemiluminescence in neutrophil stimulated by zymosan and phorbol myristate acetate, in the group of patients treated with fish oil supplementation. Moreover, in the experimental group, a reduction of Ritchie index, morning stiffness and ESR was shown [70].

In a multicenter double-blind RCT conducted in three Danish hospitals, patients were treated with a dietary supplementation of 3.6 g of n-3 PUFAs or with fat composition as the average Danish diet. Authors described an improvement in morning stiffness and TJC at week 12 [71].

In 1992, Kjeldsen-Kragh J and co-authors evaluated the effects of fish oil supplementation with or without NSAID in a double blind, clinical trial. The patients were randomized into 3 groups: in Group 1, corn oil for 16 weeks and naproxen for 10 weeks followed by a stepwise reduction to 0 mg/day during the following 3 weeks; in Group 2, EPA + DHA and naproxen for 16 weeks; in Group 3 EPA + DHA as Group 2 and naproxen as Group 1. At the end of the trial, patients in Group 2 had improved in duration of morning stiffness and global assessment by physician and patient [72].

In 1995, Kremer JM and co-authors conducted a double-blind clinical trial on active RA patients while taking diclofenac (75 mg twice a day). In experimental group a diet supplementation of 130 mg/kg/day of n-3 PUFAs was administered, while in placebo group patients took 9 capsules/day of corn oil. Placebo diclofenac was substituted at week 18 or 22, and fish oil supplements were continued for 8 weeks (to week 26). After week 26, fish oil was replaced by corn oil in both groups. At the first visit, while taking diclofenac placebo (week 22 or 26), author described an improvement in TJC, duration of morning stiffness, patient global assessment (PGA), evaluator global assessment (EGA) and physician’s evaluation of pain associated with a reduction in IL-1β levels. The decrease in the TJC remained significant 8 weeks after discontinuing diclofenac in patients taking fish oil [73]. This is a pivotal study in the field of anti-inflammatory action of n-3 PUFAs in an interesting and adequate experimental design. The results clearly demonstrated how high dose n-3 PUFAs dietary supplementation can replace and maintain NSAID clinical efficacy. The contribution of cod liver oil supplementation in reducing daily NSAID requirement of RA patients was also evaluated in a dual-centre, double-blind placebo-controlled randomized study of 9 months duration. Patients were instructed to gradually reduce, and if possible, stop their NSAID intake at week 12. A reduction by 30% in daily NSAID requirement was observed in 39% of patients treated with cod liver oil compared to 10% in placebo group [74]. This study, in an interesting experimental design, confirms previous evidences about the capacity of adequate doses of n-3 PUFAs supplementation to replace NSAID in a proportion of RA patients. Furthermore, Rajaei E and co-authors conducted a double-blind RCT to evaluate the effects of n-3 PUFAs on disease activity and remission of DMARDs treated RA. In the group supplemented with n-3 PUFAs, authors described an improvement in the PGA and EGA and a reduction in NSAID assumption [75].

In a placebo controlled clinical trial, Nordstrom DC et al. evaluated the effect of ALA on clinical and laboratory RA parameters and on EPA and DHA levels. After 3 months of dietary supplementation with ALA there were no changes in clinical, laboratory and lipid parameters in treatment group compared to placebo [76]. However, the conclusions of this study could be affected by the small sample size and by the short study duration.

The effects of fish oil in early RA, in the context of a treat-to-target strategy of DMARDs combination, were explored in a RCT by Proudman SM and co-authors. All enrolled patients started a DMARDs combination therapy including methotrexate, sulfasalazine, hydroxychloroquine and then leflunomide, according to a predetermined algorithm. Every step in the algorithm was based on disease activity and safety issues. The primary outcome measure was failure of triple DMARDs therapy. Failure of triple DMARDs therapy was lower in the fish oil group (HR = 0.24). The rate of first American College of Rheumatology (ACR) remission was significantly greater in the fish oil group (HR = 2.09) [77]. This is another pivotal study because is the first exploring the effect of fish oil supplementation in the frame of the current RA management. The study is optimally designed and the results are just impressive.

Overall, several studies evaluated the effect of n-3 PUFAs supplementation on RA patients. Most of the studies are of historical interest and present remarkable results but often are designed in the setting of an outdated RA management. However, we must stress how some of these studies, such as the ones by Kremer et al. [73] and of Galarraga et al. [74], demonstrated the ability of high doses of n-3 PUFAs supplementation to replace NSAID in a large part of RA patients. Finally, the study of Proudman SM et al. [77] clearly demonstrated the effect of n-3 PUFAs supplementation on clinimetric index used in the treat-to-target scenario.

## Clinical trials in RA: intravenous n-3 PUFAs

Leeb BF and co-authors reported an open pilot study with one group design to assess the efficacy and tolerability of intravenously applied fish oil in patients with active RA, maintaining background therapy unchanged. At the end of a 5 week follow-up, therapy was tolerated and lead to significant reduction in DAS28 from baseline [78]. The efficacy and safety of parenteral n-3 PUFAs in RA was evaluated in a double-blind, RCT in 23 patients with moderate to severe RA by Bahadori B and collegues. Intravenous infusion of fish oil emulsion or 0.9% saline was followed by 20 week of oral therapy of fish oil capsule or placebo. At the end of the study, the SJC and the TJC improved in the group treated with fish oil infusion and capsule [79]. The interest of these studies is limited by the open label and the absence of control group in the first study and by the small sample size and the short observation in the second study.

## Case control study on n-3 PUFAs and RA

Shapiro JA and co-authors conducted a population-based case-control study in women, comparing 324 incident rheumatoid arthritis cases with 1245 controls. A food frequency questionnaire to ascertain diet during a 1-year period, 5 years before a reference date (first physician visit for joint-symptoms), was used. Consumption of broiled or baked fish, but not of other types of fish, was associated with a decreased risk of rheumatoid arthritis, and this association was stronger in the rheumatoid factor seropositive subgroup. Adjusted OR for broiled and baked fish oil was 0.78 and 0.57 [80]. The author report a study with an interesting experimental design with a relevant number of RA incident cases, concluding that consumption of fish with adequate n-3 PUFAs contents could be a protective factor against RA.

Gan RW and co-authors conducted a nested case-control study to determine the association between RF and anti-CCP2 positivity and n-3 PUFAs percentage in erythrocyte membranes (RBCs). RA patients were enrolled from the Studies of the Etiology of RA (SERA) cohort including RA-free participants who are at increased risk for RA. Increasing n-3 PUFAs% in red blood cells (RBCs) was inversely associated with RF and anti-CCP2 positivity in shared epitope (SE)-positive participants (OR = 0.27 and OR = 0.42 respectively). In the SERA cohort at baseline, n-3 PUFAs supplement use was associated with a lower prevalence of RF positivity in SE-positive participants (OR = 0.32) [81].

In the same cohort, in another nested case-control study, anti-CCP2 positive cases were less likely than controls to report n-3 PUFAs supplement use (OR = 0.14). Moreover, the likelihood of anti-CCP2 positivity was inversely associated with total n-3 PUFAs% in RBCs (OR = 0.47) [82].

The interest of both of these nested case control studies is the enrollment of patients from the SERA cohort. The results underline the possible impact of n-3 PUFAs on anti-CCP2 or RF positivity.

In conclusion, case-control studies on the relation between n-3 PUFAs and RA incidence suggest the possible role of dietary n-3 PUFAs in prevention of RA incidence overall and of seropositivity development in at-risk subject with shared epitope.

## Perspective cohort study on fish oil consumption and RA

In a large perspective study linked to the Danish National Patient Registry, 57,053 individuals completed a detailed self-administered food frequency questionnaire. The average time of cohort follow-up was 5.3 years. During follow-up, 69 subjects developed incident RA. Each increase in intake of 30 g fat fish (> or = 8 g fat/100 g fish) per day was associated with 49% reduction in the risk of RA [83].

Di Giuseppe D and co-authors analysed the association between the dietary content of n-3 PUFAs and incidence of RA in woman from Swedish Mammography Cohort, a population based prospective study. A self-administered food-frequency questionnaire (FFQ) was administered. Among 32,232 women, 205 RA cases were identified during a mean follow-up of 7.5. Compared to lower intake, an intake of dietary n-3 PUFAs of more than 0.21 g/day was associated with a multivariable adjusted RR of 0.65 of developing RA. Long-term intake consistently higher than 0.21 g/day was associated with a 52% decreased risk of RA. Consistent long-term consumption of fish ≥1 serving per week compared with <1 was associated with a RR of 0.71 of developing RA [84].

Both these studies present consistent experimental design with considerable sample size and long tern observational time. They unmistakably underline the possible protective role of a diet highly rich in n-3 PUFAs against RA development.

## Meta-analysis

Goldberg RJ and Katz J conducted a meta-analysis on the 19 RCTs available in 2007 assessing the pain-relieving effects of n-3 PUFAs in patients with RA or joint pain secondary to inflammatory bowel disease and dysmenorrhea. Standardized mean differences (SMDs) as a measure of effect size were used. 3–4 months dietary supplementation with n-3 PUFAs improved clinical parameters such as patient reported joint pain intensity (SMD: −0.26), duration of morning stiffness (SMD: -0.43), TJC (SMD: -0.29), and NSAID consumption (SMD: −0.40) [85].

Lee YH and co-authors reported a meta-analysis on 10 RCTs evaluating the effects of n-3 PUFAs at doses ≥2.7 g/day for a minimum of 3 months on clinical outcome in RA. The analysis involved 183 RA patients and 187 placebo-treated patients. n-3 PUFAs reduced NSAID consumption (SMD −0.51). Other parameters, such as TJC, SJC, morning stiffness and physical function showed a trend to improve in n-3 PUFAs treated group, but they did not reach statistical significant [86].

Both this meta-analysis evaluated the effect of n-3 PUFAs supplementation on RA clinical parameters. However, the major part of the studies included were conducted in the 80s and 90s referring to an obsolete RA management. This consideration could impact on the effort of the conclusions.

The association between fish consumption and risk of RA was evaluated in a dose-response meta-analysis conducted by Di Giuseppe D and co-authors. Seven studies (four case-controls and three prospective cohorts) involving a total of 174,701 participants and 3346 cases were included in the meta-analysis. The relative risk (RR) of RA was 0.96 for each one serving per week increment in fish oil consumption. As compared with never consumption, the RR of RA was 0.76 lower to 1 up to 3 serving per week of fish [87]. This meta-analysis confirms the results seen in individual case control and cohort studies concerning the possible protective role of diet rich in fish against RA incidence.

## RA and PUFAs: beyond the joints

Patients with RA have a higher to develop osteoporosis [88]. It is, nowadays, well known how PUFAs can deeply interfere with bone metabolism, in a way which is strictly dependent on the nature of compound considered, so that a high ratio n3/n6 fatty acid diet is considered as protective from bone density loss [89]. Of interest, if n-6 PUFAs AA, but not n-3 PUFAs, is responsible for inhibiting osteoblastogenesis and inducing adipogenesis of human mesenchymal stem cells, acting as inhibitors of *opg/rankl* gene expression in osteoblasts, and consequently altering their differentiation process and promoting, by this way, the loss of bone mass [90]. Moreover, osteoclast in vitro growth and activity is far less evident in the presence of CSF and receptor activator of NF-κB ligand, if culture medium is enriched with Resolvin E1, (RvE1) which is for this reason defined as a regulator of maturation [91]. This feature is further elucidated by the ability of RvE1 to enhance expression of osteoprotegerin, a receptor for RANKL, which in turn promote the bone mass growth [92]. Clinically, it has been demonstrated how 3 months oral dietary supplementation of Conjugated Linoleic Acid (CLA) in RA patient is able to induce variation in level of IGF1, telopeptides C, and Osteocalcin [93].

It is nowadays well known how patients with RA suffer from an augmented risk of CV comorbidity and mortality. Traditional risk factors alone are not sufficient to explain this feature, which depends both on pharmacological treatment and on the chronic inflammatory state, which characterize the disease and which represent the keystone of others chronic inflammatory disorders such as atherosclerosis [94]. On the other hand, PUFAs play an important role in reducing cardiovascular risk in general population. First of all, n-3 PUFAs dietary supplementation is related to an increased endothelium-induced vasodilation [95–97], increased availability of NO, altered eicosanoid profile such as increasing in PGI_2_ and PGI_3_ [98–100]. Moreover, n-3 PUFAs effects on CV system are also evident in inhibiting arterial stiffness [101, 102], in plaques formation and modification process [103, 104], in regulation of lipid plasma profile [105, 106]. Many studies demonstrated a beneficial effect of n-3 supplementation in primary and secondary cardiovascular prevention [107]. Dawczynski C and co-authors evaluated the effect of n-3 PUFAs on cardiovascular and inflammatory parameters of RA, including immunological parameters, biomarkers of oxidative stress, serum lipids, and disease activity. They reported a double-blind RCT with a cross-over design with diet enriched with high concentration of EPA and ALA or placebo. At the end of the treatment period, the enriched diet improved serum lipids by increasing HDL and lowering lipoprotein a and decreased lipopolysaccharide-stimulated cylo-oxygenase-2 expression. The long-term consumption of n-3 PUFAs enriched dairy products (2 × 12 weeks) favored the diastolic blood pressure [108].

n-3 supplementation could represent a therapeutic frontier in order to reduce cardiovascular risk in rheumatoid arthritis, but studies on this area are still missing [109].

## Conclusions

Growing evidence demonstrates a role of PUFAs in chronic inflammation of RA and this is well established in many types of animal models of inflammatory arthritis.

In addition, several case-control and prospective cohort studies show that a high content of n-3 PUFAs in the diet could play a protective role for incident RA in subjects at risk. At present, we have no data about n-3 PUFAs enriched daily products supplementation and the incidence of the disease in high risk population.

Since the mid 80s, the attention of the scientific community has focused on the possible use of PUFAs in RA therapy. Clinical trials developed in the 80’s, 90’s and 2000 showed the ability of PUFAs dietary supplementation to significantly improve the pain symptoms (evaluated by patients and physicians), the tender joint count, the duration of morning stiffness and the frequency of NSAIDs assumption. Nevertheless, the heterogeneity of endpoints, time of observation, sample size, inclusion criteria and administered amounts of EPA and DHA results in a limitation in comparing different studies. Moreover, only recent clinical trials have focused their attention on the possible effectiveness of dietary n-3 PUFAs supplementation in early arthritis and in the setting of treat to target strategy, which represent a major challenge for Rheumatologists in recent years [1]. Many other questions still remain unanswered. First of all, we do not have any information about the effectiveness of n-3 PUFAs supplementation in combination with biologic DMARDs, which are largely used as treatment for active RA. Moreover, none of the analysis reported in this review analyzed the role of n-3 PUFAs on radiographic progression or synovial histopathology. Endocannabinoid system (eCBs) represents a valuable novel therapeutic target in inflammatory diseases and appears to be dysregulated in RA pathogenesis [110–112]. No studies have evaluated the role of n-3 PUFAs supplementation and the eCBs alterations, yet. n-3 PUFAs have not only anti-inflammatory properties, but their metabolites act as specialized pro-resolving mediators (SPMs): further analysis should focus on the efficacy of n-3 PUFAs derived SPMs in RA. More studies are needed to assess the real value of n-3 PUFAs as therapeutic options also for the management of osteoporosis and cardiovascular diseases, which are well established comorbidities of RA.

To summarize, these evidences open an interesting perspective in future research as much in prevention as in treating RA.

## Abbreviations

2-AG: 2-arachinoylglicerol
AA: Arachidonic acid
AEA: *N-*arachidonoylethanolamine
ALA: Alpha-linolenic acid
ARA: Arachidonic acid
ATX: Autotaxin
BMD: Bone mineral density
CB_1_: specific G protein-coupled type-1 cannabinoid receptor
CB_2_: specific G protein-coupled type-2 cannabinoid receptor
CCP: Cyclic citrullinated peptide
CFA: Complete Freund’s adjuvant
CIA: Collagen-induced arthritis
CLA: Conjugated linoleic acid
COX: Cyclooxygenase
CSF: Colony-stimulating factor
CV: Cardiovascular
DAS: Disease activity score
DHA: Docosaheaxaenoic acid
DMARDs: Disease-modifying antirheumatic drugs
DPA: Docosapentaenoic acid
eCBs: endocannabinoids
EGA: Evaluator global assessment
EPA: Eicosapentaenoic acid
EPO: Evening primrose oil
FFQ: Food frequency questionnaire
FOP: Fish oil preparation
GLA: Gamma linolenic acid
GPR: G protein coupled receptor
HDL: High density lipoprotein
IFN: Interferon
IGF: Insulin-like growth factor
IL: Interleukin
JAK: Janus kinase
LA: Linoleic acid
LOX: Lipoxygenase
LPA: Lysophosphatidic acid axis
LPC: Lysophosphatidylcholine
LT: Leukotrienes
MAG-DHA: docosahexaenoic acid monoglyceride
MAG-DPA: docosapentaenoic acid monoglyceride
MAG-EPA: eicosapentaenoic acid monoglyceride
MAPK: Mitogen-activated protein kinase
MCP: Monocyte chemoattractant protein-1
MMP: Matrix metalloproteinases
MuRF1: Muscle RING-finger protein-1
NAE: *N*-acylethanolamines
NF-κB: Nuclear factor kappa-light-chain-enhancer of activated B cells
NO: Nitric oxide
NSAID: Non steroidal anti-inflammatory drugs
OR: Odds ratio
PEA: *N*-plamitoylethanolamine
PG: Prostaglandins
PGA: Patient global assessment
PPAR: Peroxisome proliferator-activated receptor
PRO: Patient report outcomes
PUFAs: Polyunsaturated fatty acids
RA: Rheumatoid arthritis
RANKL: Receptor activator of nuclear factor kappa-B ligand
RBC: Red blood cells
RCT: Randomized controlled trial
RF: Rheumatoid factor
RR: Relative risk
Rv: Resolvine
SCW: Streptococcial cell wall
SE: Shared epitope
SERA: Studies of the etiology of RA
SJC: Swollen joints count
SMD: Standardized mean difference
SPM: Specialized pro-resolving mediator
STAT: Signal transducer and activator of transcription
Th: T helper
TJC: Tender joints count
TNF: Tumor necrosis factor
TX: Thromboxanes
